# Supplementary material for: Antibiotics Shift the Temperature Response Curve of Escherichia coli Growth
Source: mSystems. 2021 Jul 20;6(4):e00228-21. doi: 10.1128/mSystems.00228-21 (PMC8422994; doi:10.1128/mSystems.00228-21)
Supplement: TEXT S1 [file msystems.00228-21-s0001.pdf]

Supplemental Information

A novel temperature response model

The Briere model [1] for the temperature dependence of a trait g is given by equation

$$g(T) = \begin{cases} cT(T - T_{\min})(T_{\max} - T)^{\frac{1}{m}} & T_{\min} < T < T_{\max} \\ 0 & \text{otherwise} \end{cases} \tag{1}$$

where $T_{\min}$ and $T_{\max}$ are the minimum and maximum temperatures at which the response is nonzero and c is an arbitrary constant that modifies the height of the curve. The parameter m allows for changing the skew of the curve. In most applications, it is frequently fixed at $m = 2$ while the other parameters are fit to the data. The Briere model with $m = 2$ is often referred to as the Briere 1 model in the literature, while the model with m as a parameter is referred to as the Briere 2 model.

In this work, we introduce the *modified Briere model*, a novel temperature response model to better describe the temperature responses of *E. coli* growth in the presence of antibiotics. Our goals are to develop a flexible model that can generate a wider range of shapes of temperature response curves, and has biologically meaningful parameters. We start with the equation

$$g(T) = \begin{cases} c(T - T_{\min})^a(T_{\max} - T)^b & T_{\min} < T < T_{\max} \\ 0 & \text{otherwise} \end{cases} \tag{2}$$

where $T_{\min}$ and $T_{\max}$ are the minimum and maximum growth temperatures, respectively. The parameters $a, b, c > 0$ are positive constants that collectively determine the height and the shape of the curve. In this intial parametrization, these are arbitrary mathematical constants, but we replace them with more intuitive parameters below.

Compared to the original model, the modified Briere model is modified in two ways. First, the parameters a, b make it possible to generate a much wider range of curve shapes, including left-skewed, symmetric and right-skewed curves that change with varying steepness. This greater variety in shapes is necessary in order to fit our dataset of temperature responses in the presence of antibiotics. Second, the factor of T in the original model was removed. This factor (along with $m \neq 1$) makes the Briere model non-symmetrical/skewed as needed for temperature response curves, but it also introduces an implicit assumption that the response is always zero at 0°C. This makes the original Briere model unsuitable for organisms for which it is plausible that $T_{\min} < 0°\text{C}$ since the response will cross to negative values. As an alternative that is also non-symmetrical/skewed without this assumption, we can introduce an asymmetric number of factors of $(T - T_{\min})$ and $(T_{\max} - T)$ through the exponents a, b.

Derivation of T_{opt} for the modified Briere model

One of the main quantities of interest when looking at temperature responses is T_{opt}, the optimal temperature for growth. We have that, for $T_{\min} < T < T_{\max}$,

$$\log g(T) = \log c + a \log(T - T_{\min}) + b \log(T_{\max} - T)$$
$$\frac{d}{dT} \log g(T) = \frac{a}{T - T_{\min}} - \frac{b}{T_{\max} - T}$$

which implies

$$T_{\text{opt}} = \frac{aT_{\max} + bT_{\min}}{a + b}.$$

Thus, in the modified Briere model the optimum temperature is a convex combination of the maximum and minimum temperatures, which we can write as

$$T_{\text{opt}} = \alpha T_{\max} + (1 - \alpha) T_{\min}$$

where

$$\alpha = \frac{a}{a + b}.$$

From the result above, we can see that the optimal temperature T_{opt} in the modified Briere model can be anywhere between $T_{\min}$ and $T_{\max}$, depending on the value of $\alpha \in [0, 1]$. A value of $\alpha = 0.5$ corresponds to a symmetric curve where T_{opt} is exactly in the middle. As such, α is an intuitive and directly interpretable parameter that specifies the location of the temperature optimum relative to the minimum and maximum temperatures.

In contrast, for the original Briere model, the optimal temperature is a more complex expression

$$T_{\text{opt}} = \frac{2mT_{\max} + (m+1)T_{\min} + \sqrt{4m^2 T_{\max}^2 + (m+1)T_{\min}^2 - 4m^2 T_{\min} T_{\max}}}{4m + 2}$$

In the most commonly used case, where $m = 2$ is fixed,

$$T_{\text{opt}} = \frac{4T_{\max} + 3T_{\min} + \sqrt{16T_{\max}^2 + 3T_{\min}^2 - 16T_{\min} T_{\max}}}{10}$$

is a fixed function of the minimum and maximum growth temperatures. That is, the optimum temperature is completely determined (fixed) for fixed values of $T_{\min}$ and $T_{\max}$ for the Briere 1 model. Varying the parameter m in the Briere 2 model allows for the optimum to vary relative to $T_{\min}$ and $T_{\max}$, but at the cost of greatly changing the range of reasonable values for the parameter c (often by orders of magnitude), making parameter inference through Bayesian methods difficult.

Reparametrization of the modified Briere model

We next reparametrize the modified Briere model into a more intuitive form to aid the interpretability of its parameters. This is helpful both for understanding

the model and for choosing meaningful prior distributions for parameter estimation (see below). In particular, the model as written above has three arbitrary constants a, b, $c \geq 0$ with no clear physical/biological meaning. In this section, we replace these parameters with a different, more intuitive set of parameters.

Substitution and some algebra shows that the maximum growth

$$g_{\max} = g(T_{\text{opt}}) = c\left(\frac{a}{a+b}\right)^a \left(\frac{b}{a+b}\right)^b (T_{\max} - T_{\min})^{a+b}$$

Therefore, for $T \in (T_{\min}, T_{\max})$ we can rewrite the model as

$$g(T) = g_{\max}\left(\frac{T - T_{\min}}{a}\right)^a \left(\frac{T_{\max} - T}{b}\right)^b \left(\frac{a+b}{T_{\max} - T_{\min}}\right)^{a+b}$$

(where we replace c with $g_{\max}$, the maximum value of growth attained by the temperature curve).

Next, we replace the parameters a, b with $\alpha = \frac{a}{a+b}$ and $s = a + b$. This yields the final form of the modified Briere model:

$$g(T) = \begin{cases} g_{\max}\left[\left(\frac{T-T_{\min}}{\alpha}\right)^{\alpha} \left(\frac{T_{\max}-T}{1-\alpha}\right)^{(1-\alpha)} \left(\frac{1}{T_{\max}-T_{\min}}\right)\right]^s & T_{\min} < T < T_{\max} \\ 0 & \text{otherwise} \end{cases} \tag{3}$$

The interpretation of α (location of optimum temperature relative to minimum and maximum temperatures) and $g_{\max}$ comes from the discussion above. To find an interpretation for s, note that

$$\frac{d}{dT}\log g(T) = s\left[\alpha \log(T - T_{\min}) + (1-\alpha)\log(T_{\max} - T) - \log\left(T_{\max} - T_{\min}\right)\right]$$
$$\frac{d}{dT}g(T) = sg(T)\left[\alpha \log(T - T_{\min}) + (1-\alpha)\log(T_{\max} - T) - \log\left(T_{\max} - T_{\min}\right)\right]$$

Since s is a constant that multiplies the derivative, it determines the smoothness of the function $g(T)$ (i.e. how rapidly the derivative changes). Because of this, changing s while keeping the rest of the parameters fixed will result in curves with the same $T_{\min}, T_{\max}$ and T_{opt}. Small values of s correspond to smoother curves where growth decreases gradually from the maximum value at T_{opt}, while large values correspond to "skinnier" curves that decrease sharply (reducing the temperature breadth corresponding to half-maximum growth while leaving the range for non-zero growth intact).

With this parametrization, the modified Briere model is flexible: it can generate temperature response curves where $T_{\min}, T_{\max}, T_{\text{opt}}$ and the half-maximum range vary independently. Since the parameters $\{T_{\min}, T_{\max}, g_{\max}, \alpha, s\}$ are all biologically meaningful, biological knowledge about the parameters can be used to improve parameter inference through the use of informative prior distributions in Bayesian approaches.

Statistical model and justification of prior distributions

We parametrized the extended Briere model in terms of $\mathcal{P} = \{g_{\max}, T_{\min}, T_{\max}, \alpha, s\}$ as detailed above. To do parameter inference, we took a Bayesian approach. We first extend the deterministic extended Briere model to an explicit statistical model. Denote the ith measured optical density value (which is proportional to the number of bacteria) as y_i, and the corresponding temperature as T_i. We model the data as a nonlinear regression, given by

$$y_i|\mathcal{P}, \sigma_{T_i}, T_i \sim \text{Gamma}\left(\mu = g(T_i), \sigma = \sigma_{T_i}\right)$$

(where a Gamma distribution was chosen since growth is strictly positive). Note that the Gamma distribution is parametrized in terms of the mean and standard deviation. The mean of the distribution $g(T_i)$ corresponds to the deterministic extended Briere model. A different set of parameters was fit for each condition (antibiotic or antibiotic combination).

The observed standard deviation was clearly different at different temperatures. However, there was no clear trend as a function of T_i or y_i that was consistent across the different growth conditions. We took a hierarchical approach to model the standard deviation.

$$\begin{aligned} \sigma_{T_i}|\beta &\sim \text{halfCauchy}(\beta) \\ \beta &\sim \text{halfCauchy}(0.3) \end{aligned}$$

The halfCauchy family is commonly used in hierarchical models for variance parameters, as recommended by [2]. According to these recommendations, the scale parameter for the halfCauchy was chosen to be a plausible, but high, value for a standard deviation. The purpose for this weakly informative prior is to not have much effect in the parameter inference in the region of plausible values of the standard deviations (values around 0.3 or smaller), but for it to rule out implausible values that are much higher (which can cause problems when using uniform priors). The optical density data ranges roughly from 0 to 1, so this is a conservative upper bound.

Next, we justify the priors chosen for the parameters of the extended Briere model. We chose to use the following weakly informative priors for the temperatures $T_{\min}$and $T_{\max}$:

$$\begin{aligned} T_{\min} &\sim \text{Normal}(\mu = 12.5°\text{C}, \sigma = 12.5°\text{C}) \\ T_{\max} &\sim \text{Normal}(\mu = 47°\text{C}, \sigma = 7.5°\text{C}) \end{aligned}$$

While the priors are informative and not flat, these choices are quite conservative in a biological sense. They correspond to a prior belief that the minimum temperature for growth of *E. coli* (under the experimental conditions) is 95% likely to be in the interval [−12.5°C, 37.5°C] and that the maximum temperature for growth is 95% likely to be in the interval [32°C, 62°C]. From previous experiments, it is known that the optimal temperature for *E. coli* growth is around 37°C and that no growth is observed past approximately 46 to 50°C [3].

The means and standard deviations for the normal priors were chosen to be consistent with these previous experiments.

We chose flat priors for $g_{\max}$ and α that give equal prior weight to all possible parameter values. The limits on α are mathematical, since it is constrained to be $0 \leq \alpha \leq 1$. The upper bound chosen for the optical density $g_{\max}$ is informed by the results of previous similar growth experiments using the same equipment, which are known to virtually always give values below 1.

$$\alpha \sim \text{Uniform}(0,\,1)$$
$$g_{\max} \sim \text{Uniform}(0,\,1)$$

Lastly, the prior distribution for s was chosen to be weakly informative, placing weak constraints in $s = a + b$ to be small by penalizing very large values of s.

$$s \sim \text{halfCauchy}(20)$$

In the previous section, we showed that small values of s will result in smoother curves since it is a scaling factor for the derivative of $g(T)$. This prior can be thought as a form of regularization, with the goal of preventing overfitting. It corresponds to a prior belief that smoother curves should be preferred if possible (where the growth changes smoothly, rather than abruptly, with temperature). This gives smaller prior probability to curves where there are very abrupt changes in growth with small changes in temperature. Here, we took $s = 20$ to be a plausible, but high value of the parameter (in the original Briere model, $s = 1.5$).

Relationship between temperature response curves under antibiotics and drug-temperature interactions based on Bliss independence

In a previous study [4], we clustered antibiotics and non-optimal temperatures that have similar interactions (synergy or antagonism) with other stressors in terms of their effects on *E. coli* growth. The Bliss independence model for multiple stressors posits that, for a pair of non-interacting stressors x and y, if w_x and w_y are the corresponding growth proportions (compared to unstressed growth), the growth proportion in the presence of both stressors simultaneously will be $w_{xy} = w_x w_y$. Interactions between stressors can be defined based on deviations from the Bliss independence null model. Pairs of stressors for which $w_{xy} > w_x w_y$ are said to be *antagonistic*, pairs of stressors for which $w_{xy} < w_x w_y$ are said to be *synergistic* and pairs of stressors for which $w_{xy} \approx w_x w_y$ are said to be *additive* (or non-interacting).

Interactions between temperature and other stressors (such as antibiotics) can be visualized by comparing the temperature response curve in the presence of the antibiotic with the temperature response curve that would be expected under Bliss independence. If $g_0(T)$ and $g_a(T)$ are the temperature response

curves in the absence and in the presence of a stresor a (which is assumed to be an antibiotic, although the same derivation would apply to other stressors), and $g_a(T_{\text{opt}})$ the Bliss independence model posits that

$$\frac{g_a(T)}{g_{\max}} = \left(\frac{g_0(T)}{g_{\max}}\right)\left(\frac{g_a(T_{\text{opt}})}{g_{\max}}\right)$$

Therefore, the Bliss independence model predicts a temperature response curve when antibiotic a is present (assuming there are no drug-temperature interactions):

$$\tilde{g}_a(T) = \frac{g_0(T)g_a(T_{\text{opt}})}{g_{\max}}$$

We draw a distinction here between the predicted curve by Bliss independence (which we denote by $\tilde{g}_a(T)$) and the empirical temperature response curve under antibiotic a (which we denote as $g_a(T)$).

Deviations between these temperature response curves correspond to interactions (synergy or antagonism) in the Bliss independence framework. In particular, temperatures for which $g_a(T) < \tilde{g}_a(T)$ are *synergistic* with the antibiotic and temperatures for which $g_a(T) > \tilde{g}_a(T)$ are *antagonistic* (Supplemental Figure 7). Due to these results, the shared-damage hypothesis presented here can be given a rough interpretation in terms of Bliss interactions: stressors that damage similar cellular components will tend to have synergistic interactions to each other. Note that $\tilde{g}_a(T)$ merely scales the original temperature response curve by a factor of $\frac{g_a(T_{\text{opt}})}{g_{\max}}$, so the Bliss independence model predicts that the optimal temperature does not change. As such, shifts in the optimal temperature in the presence of a stressor imply that deviations from Bliss independence exist. Due to the geometry of temperature response curves, when this is the case the interaction type (synergy or antagonism) must change at either side of the original optimal temperature (i.e. in the absence of the stressor).

Statistics for drivers of temperature curves

Let $X_{ij} = 1$ if drug i was the driver when in combination with drug j and $X_{ij} = 0$ otherwise. For mathematical convenience, define $X_{ii} = 0$.

We do a permutation test where we randomly reassign the observed data for each drug combination to a different drug combination (without replacement). This corresponds to the null hypothesis that all drugs are exchangeable (i.e. the probability of a drug being a driver is the same for all drugs). We construct 100000 permutations of the labels. We test two different statistics:

A) Global statistic For each sample, order the drugs from most to least times it was observed as a driver. Index the $N = 12$ drugs in this order by $m = 1, \ldots, N$. We compare the observed statistic of the difference in the number of drivers

$$D = \sum_{m=1}^{N/2} \left(\sum_{j=1}^{N} X_{mj} \right) - \sum_{m=N/2+1}^{N} \left(\sum_{j=1}^{N} X_{mj} \right)$$

in the data with the 95% empirical quantile of the null distribution obtained when randomly permuting the drug labels i, j. If the observed D in the sample is greater than this value, we can reject the null hypothesis that all drugs are equally likely to be drivers.

b) Testing if specific drugs are drivers We calculate the maximum number of times that a drug is a driver in each permutation

$$M = \max_i \left(\sum_{j=1}^{N} X_{ij} \right)$$

and compare the distribution obtained with the observed values in the data. Antibiotic i is a driver more often than expected under the null model if the observed value of $\sum_{j=1}^{N} X_{ij}$ exceeds the 95% quantile for the distribution of M under the permutations.

References

[1] Briere, J.-F. Pracros, P., Roux, A.-Y. and Pierre, J.-S., 1999. A Novel Rate Model of Temperature-Dependent Development for Arthropods. *Environmental Entomology.* 28. 22-29. 10.1093/ee/28.1.22.

[2] Gelman, A., 2006. Prior distributions for variance parameters in hierarchical models (comment on article by Browne and Draper). *Bayesian analysis*, 1(3), pp.515-534.

[3] Schoolfield, R.M., Sharpe, P.J.H. and Magnuson, C.E., 1981. Non-linear regression of biological temperature-dependent rate models based on absolute reaction-rate theory. *Journal of theoretical biology*, 88(4), pp.719-731

[4] Cruz-Loya, M., Kang, T.M., Lozano, N.A. et al., 2019. Stressor interaction networks suggest antibiotic resistance co-opted from stress responses to temperature. *ISME J* 13, 12–23. https://doi.org/10.1038/s41396-018-0241-7
